# Hypoxia exacerbates intestinal injury and inflammatory response mediated by myeloperoxidase during *Salmonella* Typhimurium infection in mice

**DOI:** 10.1186/s13099-023-00586-5

**Published:** 2023-11-30

**Authors:** Qinfang Zhu, Ying Han, Xiaozhou Wang, Ruhan Jia, Jingxuan Zhang, Meiheng Liu, Wei Zhang

**Affiliations:** Research Center for High Altitude Medicine, Key Laboratory of High Altitude Medicine (Ministry of Education), Key Laboratory of Application and Foundation for High Altitude Medicine Research in Qinghai Province (Qinghai-Utah Joint Research Key Lab for High Altitude Medicine), Qinghai University, Xining, China

**Keywords:** Hypoxia, Myeloperoxidase, Intestinal mucosa, Oxidative stress, Inflammation, *Salmonella* Typhimurium

## Abstract

**Background:**

High-altitude exposure can cause oxidative stress damage in the intestine, which leads to increased intestinal permeability and bacterial translocation, resulting in local and systemic inflammation. Control of infection is critically dependent on the host’s ability to kill pathogens with reactive oxygen species (ROS). Myeloperoxidase (MPO) targets ROS in pathogens. This study aimed to investigate the effects of hypoxia on the colonic mucosal barrier and myeloperoxidase (MPO)-mediated innate immune response in the colon.

**Methods and Results:**

Genetically engineered mice were exposed to a hypobaric oxygen chamber for 3 days and an inflammation model was established using *Salmonella* Typhimurium infection. We found that hypoxic exposure caused the development of exacerbated bacterial colitis and enhanced bacterial dissemination in MPO-deficient mice. Infection and disease severity were associated with significantly increased Ly6G^+^ neutrophil and F4/80^+^ macrophage counts in infected tissues, which is consistent with elevated proinflammatory cytokines and chemoattractant molecules. Hypoxia restrained antioxidant ability and MPO deficiency aggravated the respiratory burst in the colon.

**Conclusion:**

Hypoxia can damage the colonic mucosa. MPO mediates the innate immune response and regulates the mucosal and systemic inflammatory responses to *Salmonella* infection during hypoxia.

## Background

Gastrointestinal (GI) problems are common at high altitudes, and diarrhea is frequently observed, especially among short-term visitors [1]. High altitudes may enhance susceptibility to certain pathogens, and GI infections are a major problem for mountain climbers [2]. In human and animal studies, increasing evidence suggests that exposure to hypoxic conditions impairs the intestinal barrier [3–6]. High-altitude exposure induces hypoxia and oxidative stress, which may disrupt the intestinal barrier and lead to bacterial translocation and systemic inflammation [7]. Exposure to hypoxic conditions can affect innate and adaptive immune functions [8].

Inflammation and oxidative stress are important mechanisms underlying intestinal barrier damage. Oxidative stress refers to an increased generation of intracellular reactive oxygen species (ROS), which damage DNA, lipids, and proteins and affect signal transduction [9–11]. Moreover, ROS formation is one of the hallmarks of hypoxia, which weakens the antioxidant defense system [12–14]. High levels of ROS can trigger an increase in inflammatory responses [15].

Nuclear factor erythroid 2-related factor 2 (Nrf2) is a major component of the antioxidant system and can be widely expressed in various cells and tissues [16, 17]. However, excessive oxidative stress can cause nuclear translocation of Nrf2 and the activation of downstream antioxidant proteins [18–20]. Oxidative stress enhances viral replication in some viral infections [21]. This condition plays a significant part in the pathogenesis of other infectious illnesses.

In addition, ROS can modulate the immune system [22, 23]; phagocytes use ROS production to defend against bacterial infection [24, 25]. Neutrophils use phagocyte enzyme known as NADPH oxidase to produce superoxide anions (O_2_^−^), which undergo dismutation to form hydrogen peroxide (H_2_O_2_) [26, 27]. The myeloperoxidase (MPO) enzyme can convert O_2_^−^ and H_2_O_2_ into hypohalites (predominantly HOCl) [28]. The MPO/HOCl system is critical for neutrophil-mediated microbial death and MPO alleviates collateral tissue damage during an outbreak of antimicrobial oxidation [29]. However, in contrast to NADPH oxidase, MPO has a limited function in controlling infection in either humans or mice. The important role of MPO in host innate defense is only evident when pathogen exposure exceeds the capacity of other host defenses.

The foodborne pathogen *Salmonella* Typhimurium (*S.* Typhimurium) is a Gram-negative organism that triggers the host’s innate immune response and causes acute intestinal inflammation [30]. *S.* Typhimurium is a major cause of acute gastroenteritis, a public health issue. Thus, this bacterium provides an excellent model for studying immune responses in the gut. The ability of MPO-knockout (KO) to fight against pathogens has revealed the role of this component in the host’s defense against infection. These animals exhibited increased susceptibility to some bacterial infections, such as *Candida albicans* [31] and *Klebsiella pneumoniae*, compared to wild-type (WT) mice [32]. Recent studies on MPO-KO mice revealed that this component plays a role in the development of various inflammatory conditions, such as pulmonary inflammation and atherosclerosis. However, few studies have focused on MPO in the colon; thus, the effects of hypoxia on MPO and the defense mechanisms of MPO against *S*. Typhimurium infection remain unclear.

In this study, we created conditions that were similar to a 5,000 m plateau by using a hypobaric oxygen chamber and established a mouse model of intestinal *S.* Typhimurium infection, which was used as an experimental model to investigate human intestinal diseases [33, 34]. Genetically engineered mice were used to study the function of hypoxia and MPO in host innate defense against both mucosal and systemic infection by *S.* Typhimurium as well as the mechanism of action. Our results revealed the intricate interactions between hypoxia, immune defense, MPO, and enteric pathogens.

## Results

### Hypoxia increases the morbidity of mice during ***Salmonella*** infection

**Fig. 1:**
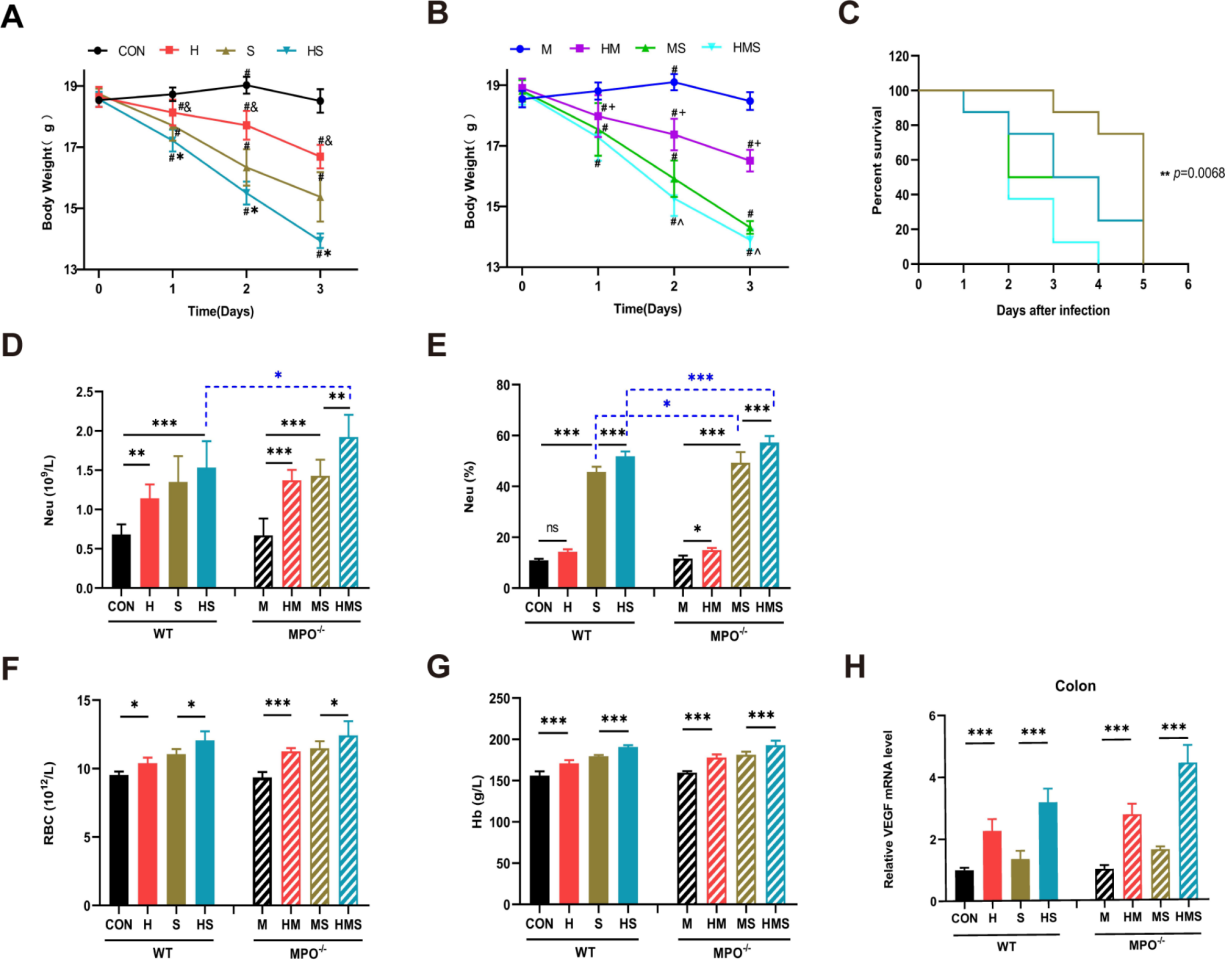
Hypoxia increases mortality in *Salmonella*-infected mice. (**A**) The changes in the body weight in WT mice. (**B**) The changes in the body weight in MPO^−/−^ mice. Data were analyzed using repeated-measures ANOVA for body weight (n = 8/group; # *p* < 0.05 versus respectively with their baseline weight; & *p* < 0.05 versus CON group at the same time; *. *p* < 0.05 versus S group at the same time; + *p* < 0.05 versus M group at the same time; ^. *p* < 0.05 versus MS group at the same time). (**C**) Mice were continuously monitored for their survival (n = 8 /group). (**D**) neutrophilic granulocytes, (**E**) percentage of neutrophils, (**F**) red blood cell, and (**G**) hemoglobin were measured at 72 h. (**H**) Gene expression of VEGF was assessed via qPCR in colon. Values are expressed as mean ± SEM (n = 8 /group) and analyzed via one-way ANOVA with Tukey’s multiple comparisons test (*p < 0.05; **p < 0.01; ***p < 0.001)

In this study, female Wild-Type (WT) mice and MPO^−/−^ mice were used as research objects to establish a simulated plateau hypoxia model and an inflammation model induced by *S*. Typhimurium. Mice were initially divided into eight groups: control group (CON), *S*. Typhimurium infection group (S), hypobaric hypoxia group (H), hypobaric hypoxia plus *S*. Typhimurium infection group (HS), MPO^−/−^ group (M), *S*. Typhimurium infection MPO^−/−^ group (MS), hypobaric hypoxia MPO^−/−^ group (HM), and hypobaric hypoxia plus *S*. Typhimurium infection MPO^−/−^ group (HMS). The body weight changes of mice in each group were monitored daily and it was found that hypoxic exposure and *S.* Typhimurium infection induced body weight loss. There was no significant difference in the baseline body weight between the WT and MPO^−/−^ groups. Compared with the gradual increase in body weight in the CON and M groups, a pronounced decrease was observed in the H and HM groups (Fig. 1A, B). The reduction in body weight of mice was significantly different from their respective baseline levels in the H and HM groups (Fig. 1A, B). As expected, mice infected with *S.* Typhimurium exhibited significant body weight loss which was pronouncedly different from their respective baseline levels (Fig. 1A, B). At the same time in the experiment (day 1, day 2, day 3), there were significant differences in body weight between H and CON, HM and M, and HS and S, while there were significant differences in body weight between HMS and MS on day 2 and day 3. Moreover, mice infected with *S.* Typhimurium (S, HS, and MS groups) exhibited high mortality (100%) on day 5 of infection. In contrast, the MPO^−/−^ mice infected with *S.* Typhimurium and exposed to hypoxia (HMS group) exhibited more significant early mortality, with 100% mortality reached at 4 days after infection (Fig. 1C). By survival analysis, *P* = 0.0068 indicates that the four groups of mice infected with *S*. Typhimurium (S, HS, MS, and HMS groups) are different overall, that is, there are differences between at least two groups. Pairwise comparison was performed among the four groups, between which HMS group and S group (*P* = 0.0008 < 0.0083), indicating a significant difference, but there is no significant difference between S and MS (*P* = 0.0382 > 0.0083) and between HS and HMS (*P* = 0.0785 > 0.0083).

The number and proportion of neutrophils in peripheral blood increased during hypoxia (Fig. 1D, E), increased significantly after infection (Fig. 1D, E), and were significantly higher in the MPO^−/−^-infected mice than in the WT-infected mice (Fig. 1D, E). These results suggest that neutrophil counts and percentages are increased in hypoxia and bacterial diseases.

Hypoxic stress induces erythrocytosis [35]. The RBC and Hb levels in the blood were monitored to verify the hypoxia model (Fig. 1F, G). Hypoxic conditions were determined by induction of vascular endothelial growth factor (VEGF) in the colon (Fig. 1H). The results showed that the red blood cell (RBC), hemoglobin (Hb), and VEGF levels increased after hypoxic exposure (Fig. 1F, G, H), which demonstrated that the hypoxic model was successfully established (Fig. 1F, G, H).

### Hypoxia exacerbates bacterial translocation in mice infected with *S.* Typhimurium

**Fig. 2:**
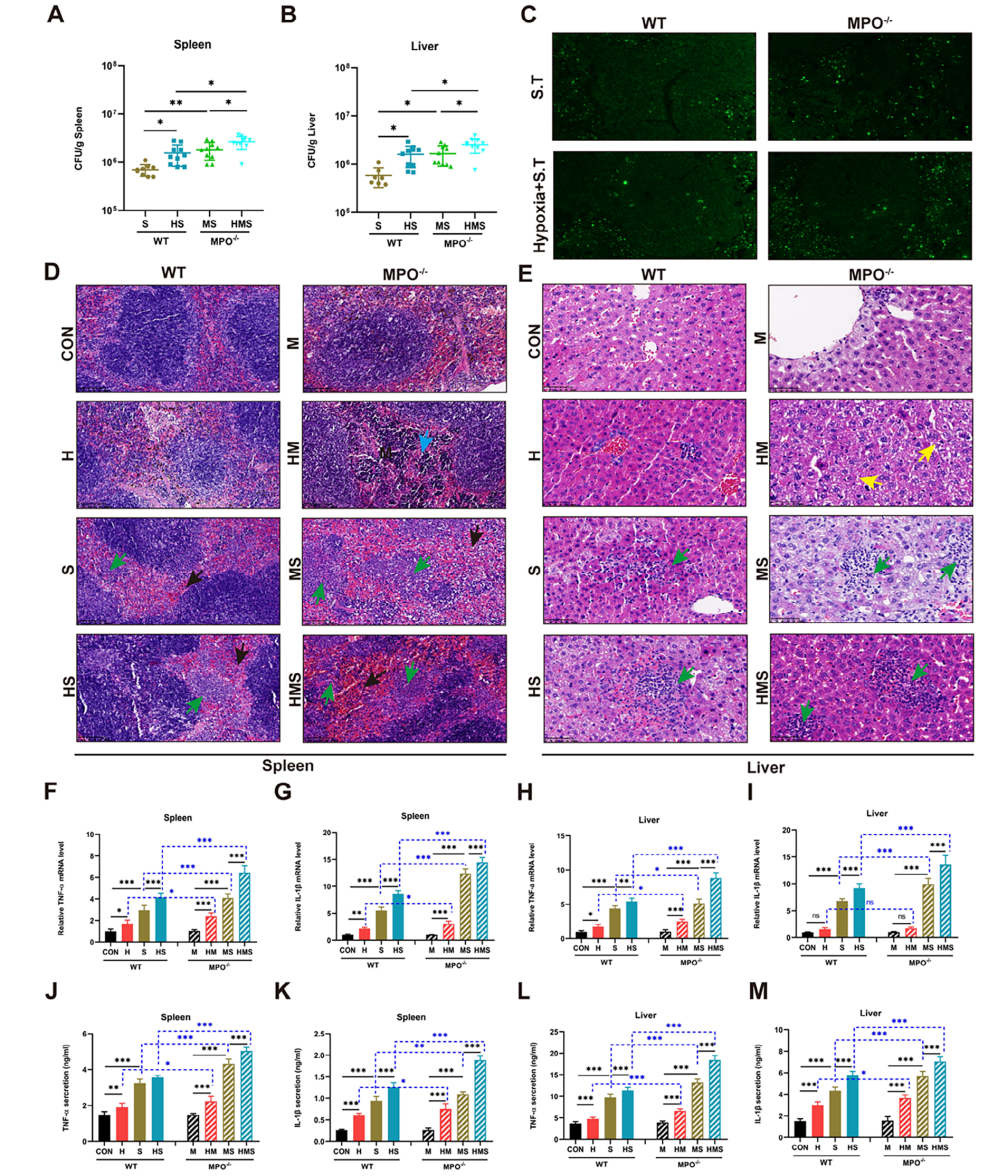
Hypoxia exacerbates bacterial translocation in *S.* Typhimurium-infected mice. Bacterial translocation to the spleen (**A**) and liver (**B**) was determined 72 h after infection via one-way ANOVA with Tukey’s multiple comparison test. The CFU number per gram of tissue was displayed (n = 8–10/group; *p < 0.05; **p < 0.01; ***p < 0.001). (**C**) Immunofluorescence microscopy revealed the presence of *Salmonella* (in green) in spleen sections (×200). The spleen (**D**) and liver (**E**) were stained with hematoxylin and eosin [(**D**) ×200; (**E**) ×400]. The blue arrow represents lymphocyte hyperplasia. The black arrow means red marrow congestion. The yellow arrow indicates that the structure of the liver plate is unclear. The green arrow represents focal abscess. (**F**–**I**) Relative mRNA expression of TNF-α (**F**, **H**) and IL-1β (**G**, **I**) in the spleen and liver tissues was detected by qPCR. (**J**-**M**) Levels of TNF-α (**J**, **L**) and IL-1β (**K**, **M**) were detected by ELISA. Data are presented as mean ± SEM (n = 8 /group) and analyzed via one-way ANOVA with Tukey’s multiple comparisons test (*p < 0.05; **p < 0.01; ***p < 0.001)

We hypothesized that high mortality is associated with sepsis. To determine whether hypoxia enhances bacterial migration and whether the increased susceptibility to *Salmonella* infection in the MPO-deficient mice is related to increased bacterial replication, we investigated bacterial translocation to the spleen and liver at 72 h after infection. No colonies were formed in the simple hypoxia group on LB plates (data not shown). The number of bacterial colonies in the spleen (Fig. 2A) and liver (Fig. 2B) of the hypoxic infection groups was significantly higher than that in the non-hypoxic infection groups (Fig. 2A, B). Moreover, the increase in the MPO^−/−^-infected mice was more pronounced than that in the WT-infected mice (Fig. 2A, B).

This was further supported by immunofluorescence microscopy analysis of the spleen tissue (Fig. 2C), which suggested that hypoxia increased bacterial translocation and MPO-deficient mice had enhanced bacterial dissemination in the spleen tissue. An increase in tissue bacterial load is accompanied by worsening inflammation and tissue damage. Histological analysis of the spleen tissue showed elevated lymphocyte levels in the HM group, indicating cellular destruction (Fig. 2D). The S and MS groups showed extensive spleen abnormalities, as evidenced by red pulp congestion, neutrophil infiltration, and abscess formation (Fig. 2D). The splenic tissue of infected mice under hypoxic condition, HS and HMS groups showed obvious hyperemia red pulp congestion, multiple small abscess foci, and splenic architecture disruption. The MPO^−/−^-infected group was more severely affected than the WT-infected group (Fig. 2D).

Concurrent with the above analyses, liver histomorphology was observed. Histological analysis showed that the hepatocytes in the HM group were disarranged and the hepatic plate was unclear (Fig. 2E). Extensive necrosis and mixed inflammatory cell infiltration were observed in both WT-infected and MPO^−/−^-infected liver sections. The liver lesions in the WT-infected mice were characterized by neutrophil infiltration and small abscess formation. MPO^−/−^-infected mice showed extensive neutrophilic infiltration with histiocytic and lobulated neutrophils, and multi-focal small abscesses. Liver lesions in WT and MPO^−/−^ mice with hypoxic infection also showed extensive neutrophil infiltration and multi-focal small abscess formation (Fig. 2E). These results suggest that there is significant inflammation and tissue damage in the spleen and liver of both WT mice and MPO^−/−^ mice after *Salmonella* infection because of the higher bacterial populations present in these tissues.

These findings were consistent with the more severe inflammatory changes in the spleen and liver. *Salmonella*-infected WT and MPO^−/−^ mice showed increased mRNA expression of proinflammatory mediators, including TNFα (Fig. 2F, H) and Il-1β (Fig. 2G, I). Hypoxia significantly increased the TNFα and Il-1β mRNA levels in the MPO^−/−^ mice compared to that in the WT mice (Fig. 2F, G, H, I). In addition, the expression of these two genes was significantly increased in the *Salmonella*-infected MPO^−/−^ mice compared to that in the WT-infected mice (Fig. 2F, G, H, I). Furthermore, the concentration of TNF-α (Fig. 2J, L) and IL-β (Fig. 2K, M) in the spleen and liver homogenates analyzed by ELISA were consistent with the expression of mRNA. These results suggest that hypoxia leads to increased production of proinflammatory cytokines, which are overexpressed due to MPO deficiency in hypoxia or infection conditions and may be attributed to the observed aggravated tissue damage. These findings indicate that oxygen and MPO play a vital role in host resistance and survival during *Salmonella* infection.

### Hypoxia enhances macrophage and neutrophil recruitment in *S.* Typhimurium-infected mice

**Fig. 3:**
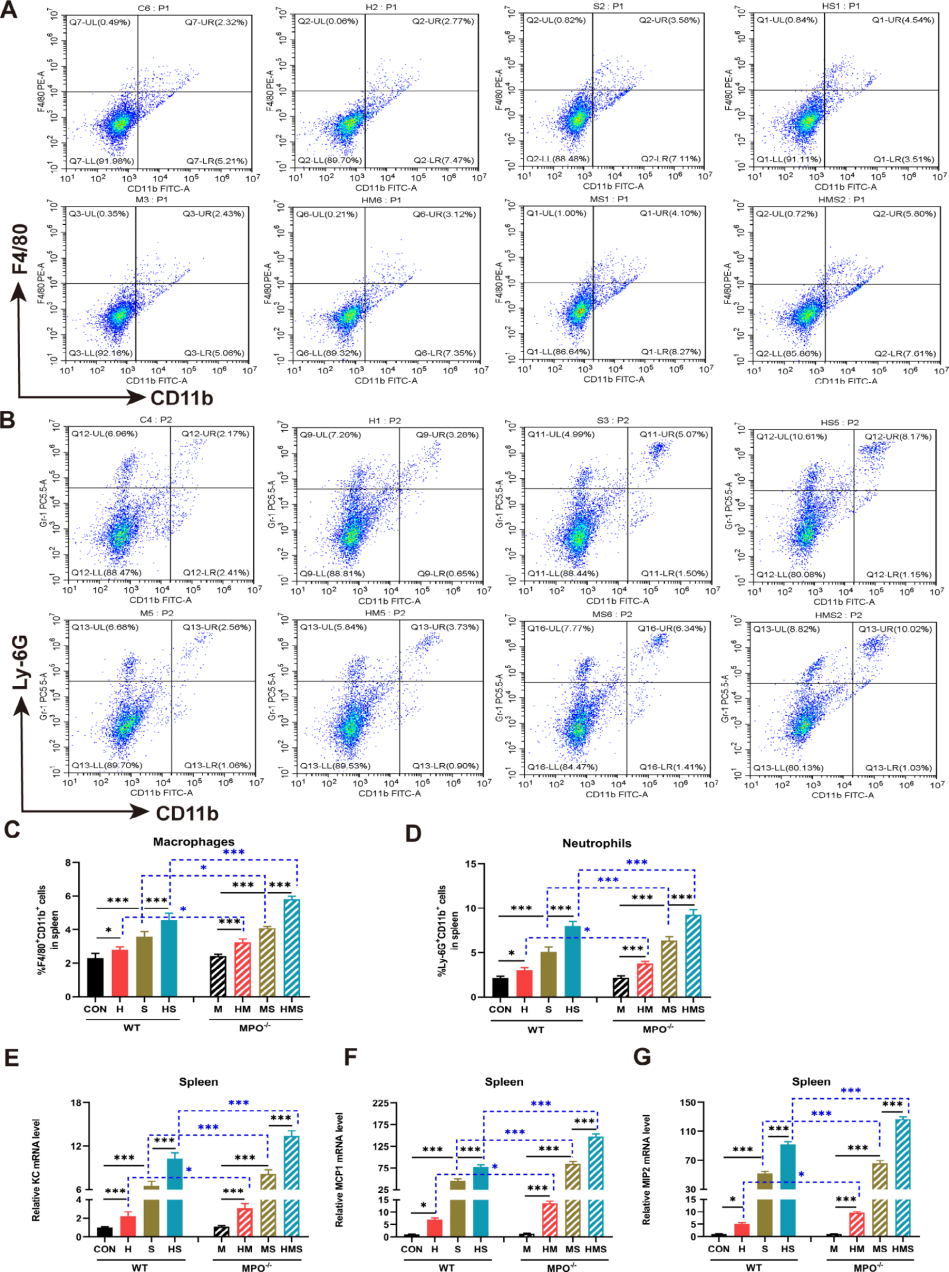
Hypoxia enhances neutrophil and macrophage recruitment in mice during *Salmonella* infection. Single-cell suspensions of spleens were prepared and stained with anti-CD11b and anti-F4/80 antibodies for macrophages (**A**) and with anti-CD11b and anti-Ly6G for neutrophils (**B**). The percentages of CD11b^+^F4/80^+^ macrophages (**C**) and CD11b^+^ Ly6G^+^ neutrophils (**D**) are shown as mean ± SEM (n = 6/group). (**E**-**G**) Gene expression of chemoattractant (KC, MCP1, and MIP2) in spleen was analyzed via qPCR (n = 8/group). Significance was analyzed by one-way ANOVA with Tukey’s multiple comparison test (*p < 0.05, **p < 0.01, ***p < 0.001)

To further study the effects of hypoxia and MPO on the innate immunity against *Salmonella* infection, we analyzed the spleen tissue’s frequency of neutrophils and macrophages. FACS analysis revealed that the spleen cells had a high number of macrophages that expressed CD11b^+^ F4/80^+^ in the MPO^−/−^ mice compared to that in the WT mice exposed to hypoxic conditions (Fig. 3A, C). Compared with WT-infected mice, the number of macrophages expressing CD11b^+^ F4/80^+^ was increased in MPO^−/−^-infected mice, and the number of macrophages expressed in MPO^−/−^-infected mice was significantly increased in comparison to the number in WT-infected mice under hypoxic conditions. (Fig. 3A, C). The frequency of CD11b^+^ Ly6G^+^ neutrophils in the spleens of the mice was consistent with the number of macrophages (Fig. 3B, D). These data suggest that hypoxia and MPO deficiency promote the migration of neutrophils and macrophages to the infection site. Next, we determined the expression of three chemoattractant molecules ((KC (CXCL1), MCP1 (CCL2), and MIP2 (CXCL2)) that regulate neutrophil and macrophage recruitment in spleen tissue. RT-qPCR results showed the hypoxia-induced upregulation of KC, MCP1, and MIP2 gene expression, and their expression in the MPO^−/−^ mice was significantly higher than that in the WT mice (Fig. 3E, F, G). *Salmonella* infection augmented the expression of these genes (Fig. 3E, F, G). The increase in chemokine expression was most apparent in MPO-deficient mice compared to the WT mice after infection (Fig. 3E, F, G). These results suggest that hypoxia and MPO deficiency may promote the recruitment of phagocytes to the site of infection by regulating the expression of chemokines, and that excessive aggregation of granulocytes in the spleen may enhance the inflammatory response during *Salmonella* infection.

### Hypoxia augments *Salmonella*-induced mucosal injury in mice

**Fig. 4:**
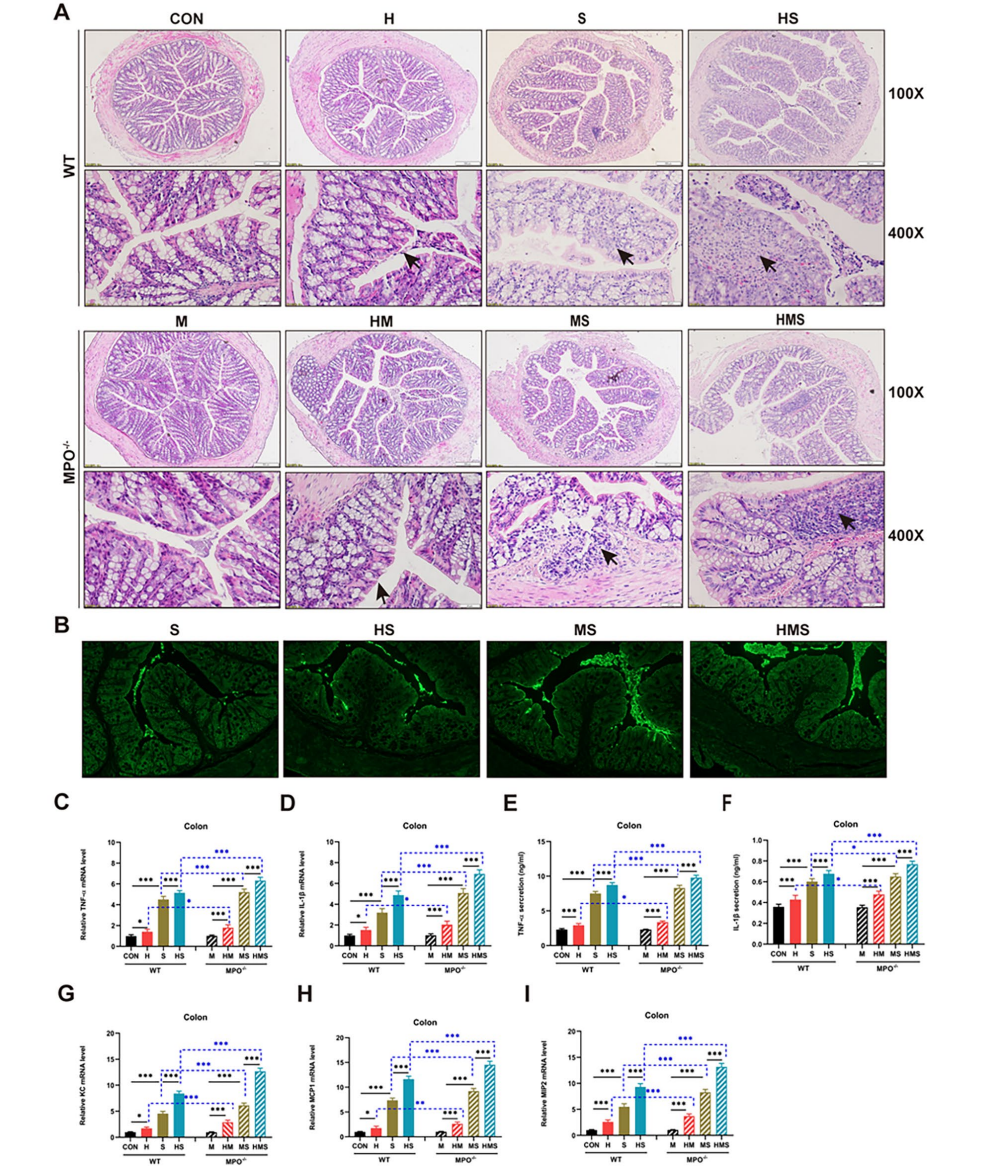
Hypoxia augments *Salmonella*-induced mucosal injury and colitis in mice. (**A**) Hematoxylin and eosin staining of the colon (×100, ×400). (**B**) Immunofluorescence microscopy data show the distribution of *Salmonella* (in green) in colon sections (×200). Levels of TNF-α (**C**) and IL-1β (**D**) were determined by quantitative PCR. Cytokine levels of TNF-α (**E**) and IL-1β (**F**) were detected by ELISA. Colon levels of KC (**G**), MCP1 (**H**), and MIP2 (**I**) were analyzed by qPCR. Data represent mean ± SEM (n = 8 /group) and one-way ANOVA followed by Tukey’s multiple comparisons test (*p < 0.05; **p < 0.01; ***p < 0.001)

Bacterial translocation is generally the result of impaired gut integrity. Our results suggest that hypoxia induces more severe liver and spleen infections in MPO-deficient mice during *Salmonella* infection. We investigated whether hypoxia and MPO levels affect innate immunity and inflammation in the intestinal mucosa. Under a light microscope, colon morphology was examined, and the results showed that the colonic mucosa had an intact epithelium and a smooth appearance in the CON and M groups (Fig. 4A). Compared with the above two groups, the recesses of the colon in the H and HM groups were shallower and flatter. Inflammatory cell infiltration was observed in the S and MS groups (Fig. 4A). The HS group showed obvious inflammatory cell infiltration and necrosis; however, this was more severe in the HMS group. Inflammatory cell infiltration with suppuration was observed in the colon of the HMS group (Fig. 4A). Immunofluorescence microscopy analysis of infected colons revealed an increased bacterial load in hypoxia-infected (Fig. 4B) and MPO-deficient mice compared to that in the WT mice (Fig. 4B). In addition, consistent with intestinal injury, hypoxia increased the levels of TNF-α (Fig. 4C) and IL-β (Fig. 4D) in the MPO^−/−^ mice compared to those in the WT mice. The mRNA expression levels of TNF-α (Fig. 4C) and L-1β (Fig. 4D) were significantly increased in the colon of MPO^−/−^-infected mice compared to that in the WT-infected mice (Fig. 4C, D). ELISA analysis of cytokine concentrations in the colon tissue was consistent with those of mRNA expression (Fig. 4E, F).

Next, we measured chemokine mRNA levels in the colon tissue. The results showed that hypoxia boosted the expression of KC, MCP1, and MIP2 in the colons of mice, especially in the MPO^−/−^ mice (Fig. 4G, H, I). *Salmonella* infection promoted chemokine expression in colon tissue (Fig. 4G, H, I), and the upregulated chemokines were significantly different in both WT-infected and MPO^−/−^-infected mice. Compared with WT-infected mice, the expression of chemokines (KC, MCP1, and MIP2) in MPO^−/−^-infected mice was more significant (Fig. 4G, H, I). These results suggest that *Salmonella*-induced inflammation can be demonstrated in both WT mice and MPO^−/−^ mice which can be exacerbated by hypoxia. Moreover, MPO^*−/−*^ mice present aggravated *Salmonella*-induced inflammation.

### Hypoxia aggravates colonic oxidative stress injury in MPO-deficient mice

**Fig. 5:**
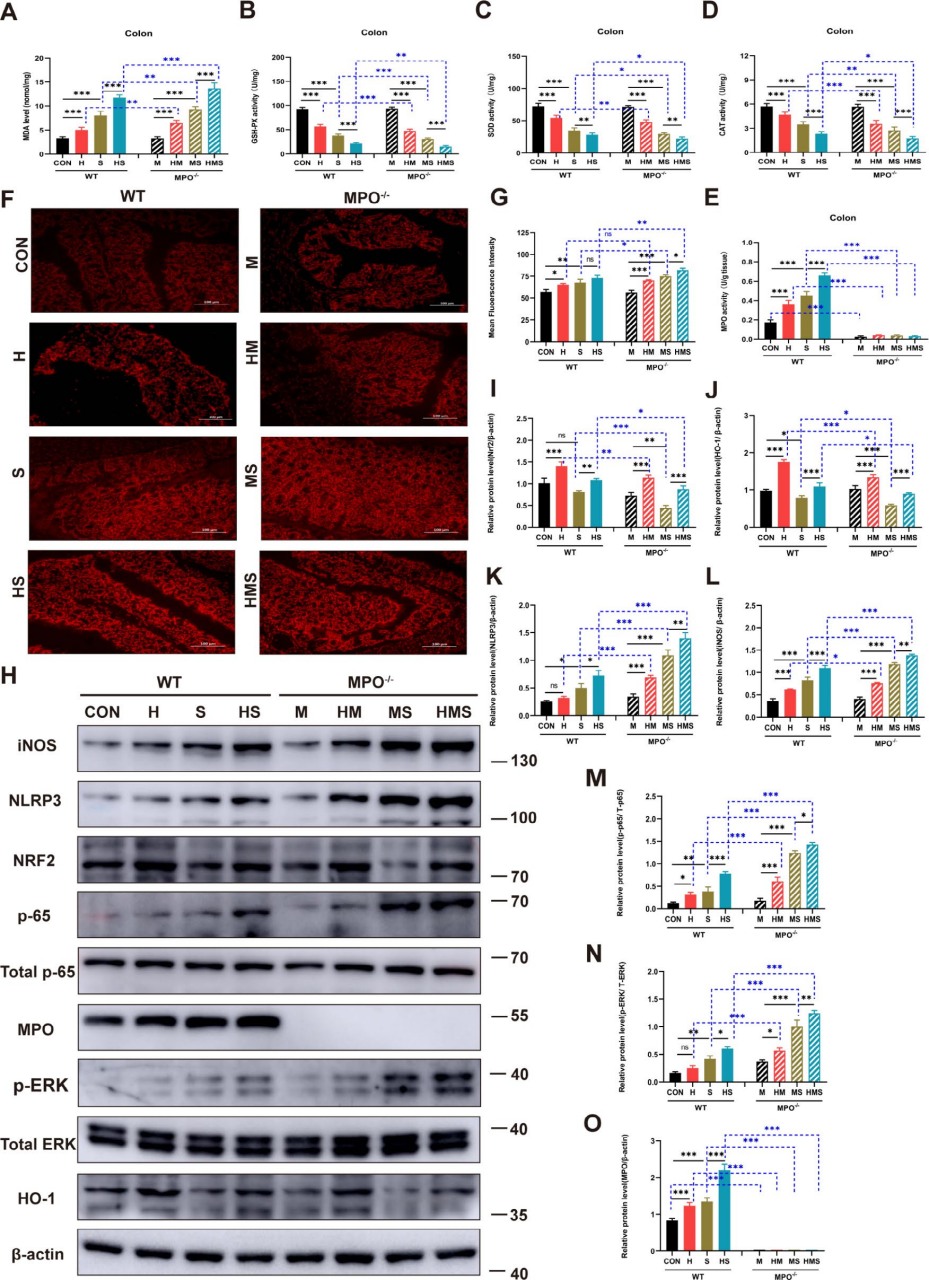
Hypoxia aggravates oxidative stress injury in colon during *Salmonella* infection Colon activity of MDA (**A**), GSH-Px (**B**), SOD (**C**), CAT (**D**), and MPO (**E**) were determined (n = 8 /group). (**F**-**G**) Mean fluorescence intensity of ROS was determined (×200) (n = 3/group). (**H**) Western blot performed and (**I**-**O**) quantification (n = 3/group). Data are presented as mean ± SEM and analyzed via one-way ANOVA with Tukey’s multiple comparisons test (*p < 0.05; **p < 0.01; ***p < 0.001)

Oxidative stress is associated with the progression of various inflammatory diseases of the colon, and the lack of antioxidant defenses in the intestinal mucosa is an essential factor leading to the impairment of the intestinal mucosal barrier [36, 37]. MPO activity is an inflammatory biomarker. Therefore, the activities of malondialdehyde (MDA), catalase (CAT), glutathione peroxidase (GSH-Px), superoxide dismutase (SOD), and MPO were measured in the colon tissue. The results showed that the activity of MDA was significantly increased and the activities of CAT, GSH-Px, and SOD were remarkably decreased under hypoxic conditions (Fig. 5A, B, C, D). The findings for MPO^−/−^ mice differed significantly from those of WT mice under hypoxic conditions (Fig. 5A, B, C, D). The activities of MDA, CAT, GSH-Px, and SOD in colon tissue after *Salmonella* infection were similar to those under hypoxia (Fig. 5A, B, C, D). Moreover, the MPO^−/−^-infected mice showed higher MDA activity but lower CAT, GSH, and SOD activities than the WT-infected mice (Fig. 5A, B, C, D). The level of intestinal MPO was increased in the WT mice under hypoxic conditions and increased significantly after infection (Fig. 5E). Next, a ROS assay was performed on frozen sections of colon tissue. The results showed that hypoxia and infection increased the mean fluorescence intensity of ROS (Fig. 5F, G). ROS production in the *Salmonella*-treated MPO^−/−^ mice was higher than that in the *Salmonella*-treated WT mice (Fig. 5F, G). The results suggest that hypoxia increases ROS levels, and MPO deficiency aggravates oxidative stress.

The protein expression levels of key molecules in the Nrf2/HO-1 pathway were determined. The protein levels of Nrf2 and HO-1 were significantly increased under hypoxia, especially in WT mice (Fig. 5H, I, J). After *Salmonella* infection in WT mice, the HO-1 protein levels decreased significantly, and the Nrf2 protein was decreased but not significantly (Fig. 5H, I, J). The protein levels of Nrf2 and HO-1 were significantly lower in the *Salmonella*-infected MPO^−/−^ mice compared to those in the WT-infected mice (Fig. 5H, I, J). In addition, the *Salmonella*-treated WT and MPO^−/−^ mice presented increased levels of proteins, including iNOS, NLRP3, p-65/T-NF-kB p65, and p-ERK/T-ERK (Fig. 5H, K, L, M, N). Hypoxia significantly increased these levels in the MPO^−/−^ mice compared to those in the WT mice (Fig. 5H, K, L, M, N). Moreover, these protein levels were significantly increased in the *Salmonella*-infected MPO^−/−^ mice compared to those in the WT-infected mice (Fig. 5H, K, L, M, N). In WT mice, the protein expression level of MPO was significantly increased under hypoxia, and *Salmonella*-infected mice showed increased protein levels (Fig. 5H, O).

## Discussion

Low-pressure hypoxic environments are observed at high altitudes, and acute hypoxia causes damage to many organs. In recent years, the damage to the human GI tract caused by high altitudes has aroused concern.

In this study, we determined the functional role of hypoxia and MPO in controlling systemic and intestinal mucosal *Salmonella* infections and the interaction between the two. First, we established a mouse model of intestinal *S.* Typhimurium infection under hypoxic conditions. The mice experienced significant weight loss and damage to the colonic mucosa. Consistent with our previous research, hypoxia causes changes in immune function in the intestine and leads to intestinal injury [38, 39]. Our study showed that hypoxic exposure significantly increased mortality in *Salmonella*-infected mice, with even higher values in the MPO-deficient mice. This is evidenced by the higher bacterial loads in the liver and spleen, which supports the hypothesis that hypoxia promotes bacterial migration. In addition, the lack of MPO resulted in impaired antibacterial activity [40], leading to the infiltration of invading bacteria into the intestinal mucosa and the transport of large amounts of bacteria and endotoxins via the blood [41] to other tissues or organs. This causes a cascade of inflammatory responses that lead to various organ dysfunction [42, 43] in distant organs and ultimately leads to death. In a sepsis model, MPO-KO mice showed marked hypothermia and high mortality after lipopolysaccharide (LPS) injection [44].

Moreover, hypoxic exposure induced the recruitment of Ly6G^+^ neutrophils and F4/80^+^ macrophages, and MPO deficiency led to a marked increase in the recruitment of polymorphonuclear neutrophils (PMN) in the infected tissues. However, the effects of hypoxia on PMN chemotaxis remain controversial. Reports have shown that the secretion of monocyte chemoattractant protein-1 (MCP-1) by fibroblasts under hypoxic conditions induces the chemotaxis of monocytes [45]. In contrast, hypoxia can inhibit MCP-1-induced migration of THP-1 monocytic cells and human macrophages [46] and inhibit MCP-1 expression and monocyte migration in ovarian cancer cell lines [47]. The different effects of hypoxia on cell recruitment may be related to the different tissues studied and the degree and duration of exposure to hypoxia. As a driving force of the inflammatory process, neutrophils are the first line of defense against the innate immune response and produce antimicrobial peptides, ROS, cytokines, and other inflammatory mediators. However, excessive neutrophil accumulation and prolonged neutrophil activation can have detrimental effects on tissues. Similarly, the excessive activation of macrophages can cause tissue damage.

Our results showed that MPO deficiency in mice significantly increased *Salmonella*-induced recruitment of neutrophils and macrophages into the colon tissue, which was associated with elevated expression of KC, MCP1, and MIP2 in the infected tissues. Similar observations have been made in recent study [48]. The researchers discovered that MPO-KO mice were more prone to experiencing severe lung inflammation exposed to *C. albicans* compared to that in WT mice and these results are associated with higher chemokine production and higher concentrations of proinflammatory factors in the lung tissue. Thus, the severity of infection is related to the excessive aggregation of chemokines and the associated induction of granulocytes.

Our results also showed that ROS production increased in the colon tissue under hypoxia, which was more obvious in the MPO-deficient infected mice and caused colon tissue injury. This observation is consistent with the finding that high-altitude exposure reduces visceral perfusion and blood oxygen levels, thereby causing hypoxia and oxidative stress [7]. These stressors may disrupt the intestinal barrier, which leads to bacterial translocation and local or systemic inflammatory responses. In critically ill patients in intensive care units, some patients, such as trauma, acute lung injury (ALI), and acute respiratory distress syndrome (ARDS), exhibit elevated ROS levels and heightened inflammatory responses due to gut-derived infections, these changes lead to multiple organ failure and death [49].

We noted that the levels of HO-1 and Nrf2 expression in the colon significantly increased when the mice were exposed to hypobaric hypoxia. Antioxidant defense factors, such as MDA, were significantly increased, whereas GSH-Px, SOD, and CAT levels were significantly decreased. Nrf2 is a factor that plays an important role in protecting cells against oxidative stress and inflammation. The effect of hypoxia on the Nrf2/HO-1 pathway remains inconsistent and controversial. Recent studies have shown that the effects of hypobaric hypoxia on the retina can decrease the expression of Nrf2/HO-1 [50]. Reports also stated that short-term exposure to hypobaric hypoxia can increase the Nrf2-dependent pathway’s regulation in rat brains [51]. Various complex mechanisms dynamically regulate the Nrf2/HO-1 signaling pathway. In the colon, the upregulation of Nrf2/HO-1 could be a compensatory response that occurs after hypoxia; however, the real antioxidant defensive ability is poor. Although acute exposure to hypoxia can enhance the innate immune response, long-term exposure to hypoxia can lead to immunosuppression [2, 52]. Therefore, the body’s innate immune system is a double-edged sword due to the possibility that too low or too high levels of immunity can damage it.

Neutrophils and monocytes produce a more intense oxidative burst than macrophages [28, 53], and neutrophils and monocytes usually express MPO; thus, they are primarily affected by MPO deficiency [53]. The absence of MPO during infection can lead to the development of detrimental effects on host tissues, such as DNA oxidation and lipid peroxidation. MPO-deficient neutrophils accumulate large quantities of H_2_O_2_; however, most H_2_O_2_ leaks from phagosomes. A study conducted on human neutrophils revealed that the levels of MPO activity and the release of extracellular H_2_O_2_ were negative after *Salmonella* stimulation [29]. Although H_2_O_2_ isn’t as reactive as HOCl, it still has strong negative effects on different biomolecules due to its oxidant properties. Indeed, MPO-deficient rodents also exhibited higher levels of H_2_O_2_ within their tissues, which further exacerbated the damage caused by *Salmonella* to host components. Therefore, *Salmonella* infection cannot be effectively controlled, which causes damage to the intestinal mucosal barrier function and aggravates local and systemic inflammatory reactions.

Increasing evidence indicates that neutrophils can mediate cell signaling by producing ROS [54]. Both the TLR ligand LPS [55, 56] and the inflammasome component NLR NLRP3 activate inflammatory cytokines by stimulating ROS production [57–59]. The enhanced ROS production that is linked to the NF-κB pathway can also cause oxidative stress [60]. Along with mediating cell communication, the ERK/NF-κΒ pathway also plays a vital role in regulating the production of chemokines and proinflammatory cytokines [61, 62]. INOS has been associated with various inflammatory bowel diseases. Reports have also shown that the development of gut barrier failure and the bacterial translocation caused by endotoxin-induced NO production is linked to iNOS upregulation [63, 64]. In our study, NLRP3 and iNOS proteins in WT mice were increased under hypoxic conditions, while ERK, NF-KB, NLRP3, and iNOS in MPO-KO mice were significantly elevated in response to hypoxia. These results suggest that hypoxia promotes inflammation in the colon of WT mice and MPO-KO mice. In MPO-KO mice by activating iNOS, the inflammasome pathway, and the ERK/NF-κB signaling pathway and enhancing inflammatory cytokine expression, thereby aggravating the breakdown of the epithelial mucosal barrier.

**Fig. 6:**
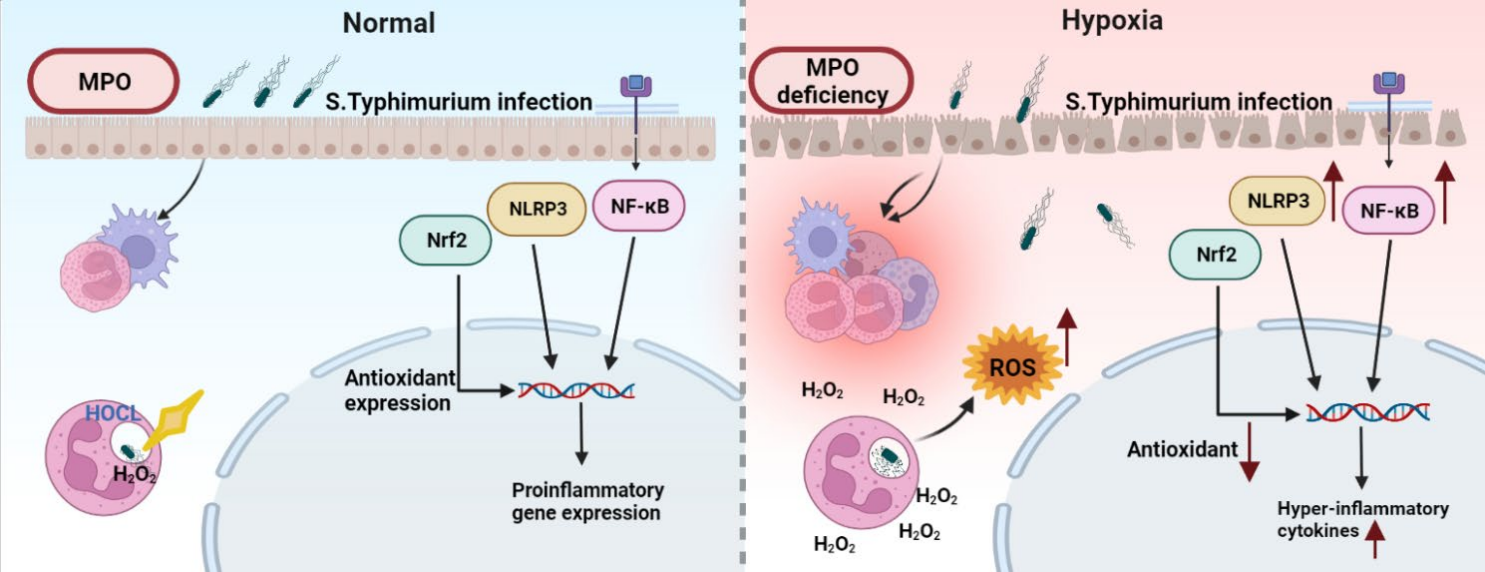
Hypoxia aggravates inflammatory response in MPO-deficient mice during *Salmonella* infection. Under the condition of *Salmonella* infection with normal MPO function, MPO played a bactericidal role. Under hypoxic exposure and MPO-deficient conditions, massive H_2_O_2_ releases and massive recruitment of neutrophils and macrophages lead to a dramatic increase in ROS, causing decreased oxidative capacity and overexpression of inflammatory genes

Our results on hypoxia-induced intestinal mucosal injury were consistent with those of previous studies. MPO is essential for the maintenance of innate immunity and epithelial homeostasis in the gut. Therefore, MPO deficiency under hypoxic conditions may be an important cause of hypoxia-induced colonic mucosal injury (Fig. 6).

## Conclusions

In summary, our results showed that hypoxia significantly damaged the colonic mucosal epithelium through oxidative stress injury, aggravated bacterial colitis, and caused bacterial translocation and systemic inflammation. Moreover, the MPO-dependent oxidative system is important in protecting host tissues from bacteria. The link between MPO oxidation system and disease is complex. Over- and under-expression of MPO can lead to worse outcomes. Understanding the interactions between various components, including hypoxia, MPO, immune function, and enteric pathogens, can provide us with valuable information on developing new treatment strategies.

## Materials and methods

### Animals

Female C57BL/6 WT mice between six to eight weeks old were obtained from the SJA Laboratory (Hunan SJA Laboratory Animal Co., Ltd., Hunan, China). MPO-KO mice were purchased from Cyagen Biosciences, Inc. (Guangzhou, China). The animals were housed in a cage within an Individual Ventilated Cage Animal Experiment System (H6, Su Hang Technology Equipment Co., Ltd., Suzhou, China) at 22 ± 2℃, with a 12 h light/ dark cycle with free access to food and water. Animal care and use conformed to the protocols approved by the Institutional Animal Care and Use Committee of Medical College of Qinghai University (Xining, China).

### *Salmonella* Typhimurium infection and hypoxia treatment

Following a week of adaptation, mice were initially divided into eight groups: control group (CON), *S.* Typhimurium infection group (S), hypobaric hypoxia group (H), hypobaric hypoxia plus *S.* Typhimurium infection group (HS), MPO^−/−^ group (M), *S.* Typhimurium infection MPO^−/−^ group (MS), hypobaric hypoxia MPO^−/−^ group (HM), and hypobaric hypoxia plus *S.* Typhimurium infection MPO^−/−^ group (HMS). *S*. Typhimurium colitis was induced by oral gavage with 3 × 10^8^ CFU of the SL1344 strain of *S*. Typhimurium, which is a streptomycin-resistant strain.

The H, HM, HS, and HMS groups were placed in a hypobaric chamber (DYC-300; Guizhou Feng Lei Oxygen Chamber Co., Ltd., Guizhou, China) to simulate an altitude of 5,000 m. The pressure was 52.9 KPa, and the CO_2_ concentration was 1,446 ppm. There were two batches of experimental animals, molded on the same day. One batch of experimental animals (CON, H, S, HS, M, HM, MS, and HMS groups) were mainly collected materials, and the experiment lasted for 3 days. Mice were weighed daily. On the morning of the fourth day of the experiment, the first mice were anesthetized with isoflurane in an induction chamber, after successful general deep anesthesia, eyeball blood was collected once. After blood collection, the mice were euthanized and fresh liver, spleen, and colon tissues were collected for further analysis. Another batch (S, HS, MS, and HMS groups) underwent a survival experiment and was observed for five days. The infected mice were subjected to survival experiments, after 5 days, and the non-dead mice were euthanized on the morning of the sixth day. Animal care and experimental protocols were performed following the National Institutes of Health (NIH) guidelines for the care and use of animals during experimental procedures. Approval was obtained from the Institutional Animal Care and Use Committee of Qinghai University, China.

### Analysis of blood samples

Blood samples were analyzed to check the levels of neutrophilic granulocytes (Neu), percentage of neutrophils (Neu%), hemoglobin (Hb) content, and red blood cell (RBC) count by Fully Automatic Blood Cell Analyzer (BC-5000; Mindray, Beijing, China).

### Histopathology

After necropsy, tissue samples of the spleen, liver, and colon, fixed with 4% paraformaldehyde, were embedded in paraffin and cut into 5 μm slices. Hematoxylin and eosin (H&E) staining was then performed. The slices were examined using an Olympus BX53 microscope (Olympus, Tokyo, Japan), and images were captured using Olympus cellSens Entry 1.14 software (Olympus).

### Immunofluorescence microscopy

Spleen and colon tissue samples were collected, frozen in Tissue-Tek® OCT compound (Sakura, Queens, NY, USA), and stored at − 80 °C. Then, 5 μm sections were cut on a Leica CM1950 Freezing Microtome (Leica Biosystems, Wetzlar, Germany). Frozen sections of tissue were fixed in acetone, washed, and blocked with bovine serum albumin (Sigma-Aldrich, St. Louis, MO, USA; V900933). Tissue cryosections of the mouse colon and spleen were incubated with *Salmonella* rabbit antibody (Thermo Fisher Scientific, Waltham, MA, USA; PA1-20811) and fluorescein goat anti-rabbit IgG (H + L) (Thermo Fisher Scientific; F-2765). The distribution of *Salmonella* in the colon and spleen was observed by microscopy (Leica Biosystems; DFCDM4 B), and images were captured using Leica Application Suite X software (Leica Biosystems).

### Measurement of bacterial translocation

Spleens and livers were collected, weighed, homogenized, inoculated on Luria–Bertani (LB) plates containing 50 µg/ml streptomycin [65], and placed in an incubator at 37℃ for 24 h to quantify the CFU value by observing colony counts.

### RNA isolation and quantitative real-time PCR (qRT-PCR)

Total RNA was extracted from the spleen, liver, and colon tissues using TRIzol reagent (Invitrogen, Waltham, MA, USA; 15,596,026). All RNA samples were reverse-transcribed to cDNA using a PrimeScript RT Reagent Kit (Takara Bio, Inc. Shiga, Japan; RR047A). Real-time quantitative polymerase chain reaction (RT-qPCR) was performed using TB Green Premix Ex Taq II (Takara Bio; RR820A) on a C.F.X. 96 Real-Time System (Bio-Rad Laboratories, Hercules, CA, USA). The primers are listed in Table 1. The data were normalized to β-actin expression and relative quantification was calculated using the 2^−ΔΔCT^ method.

**Table 1:** Information on Primer Sequences

Gene	Forward primer	Reverse primer
TNF-α	CCCTCACACTCAGATCATCTTCT	GCTACGACGTGGGCTACAG
IL-1β	TCCAGGATGAGGACATGAGCAC	GAACGTCACACACCAGCAGGTTA
VEGF	ACATTGGCTCACTTCCAGAAACAC	TGGTTGGAACCGGCATCTTTA
KC	CCGAAGTCATAGCCACACTCAA	GCAGTCTGTCTTCTTTCTCCGTTAC
MCP1	AGCAGCAGGTGTCCCAAAGA	GTGCTGAAGACCTTAGGGCAGA
MIP2	GCAGTCTGTCTTCTTTCTCCGTTAC	GCGTCACACTCAAGCTCTG
β-actin	CATCCGTAAAGACCTCTATGCCAAC	ATGGAGCCACCGATCCACA

### ELISA analysis

Liver, spleen, and colon homogenates were prepared for cytokine measurements using ELISA. IL-1β was measured by a Mouse IL-1β ELISA Kit (Elabscience, Houston, TX, USA; E-EL-M0037c), according to the manufacturer’s protocols. ELISA capture antibodies (BD Cat. #557,516) and biotinylated secondary antibodies for TNF-α (BD Cat. #558,415), and standard curve was constructed using recombinant murine TNF-α (BD Cat. #554,589) antibodies purchased from BD Biosciences (Franklin Lakes, NJ, USA).

### Flow cytometry

Single-cell suspensions of the spleen were prepared 72 h after starting the experiment. Cells were stained with anti-mouse CD11b antibody (BioLegend, San Diego, CA, USA; 101,206), Ly6G (BioLegend; 108,410), and F4/80 (BioLegend; 123,110) and then analyzed using flow cytometry (Beckman Coulter, Brea, CA, USA; A00-1-1102).

### Determination of ROS

Frozen colon tissue sections were recovered at 22 ± 2℃, and a spontaneous fluorescence quenching agent was added. The cells were then stained with a ROS dye (Sigma-Aldrich; D7008) and DAPI. The results were observed with a fluorescence microscope, and Leica Application X software (Leica Biosystems) was utilized for capturing images.

### Measurement of oxidative stress markers and MPO activity

Supernatants from colon tissues were obtained, and the protein content was determined using a BCA Assay Kit (Servicebio Co., Ltd., Wuhan, China; Cat #G2026-1000 T). Specific kits (Nanjing Jiancheng Bioengineering Institute, Nanjing, China) were utilized for analyzing malondialdehyde (MDA), glutathione peroxidase (GSH-Px), superoxide dismutase (SOD), catalase (CAT), and MPO activities according to the manufacturer’s instructions.

### Western blotting

Colon tissues were obtained and homogenized to extract the protein, which was quantified using a BCA Protein Assay Kit (Thermo Fisher Scientific; A53225). The same amount of protein was separated using sodium dodecyl sulfate-polyacrylamide gel electrophoresis and transferred onto nitrocellulose membranes (Thermo Fisher Scientific; 88,518). Membranes were blocked in 5% skim milk for 1 h and then incubated at 4 °C overnight with specific primary antibodies: anti-β-actin (1:1000, ab115777), anti-HO-1 (1:2000, ab52947), anti-ERK (1:10000, ab184699), anti-p-ERK (1:1000, ab201015), anti-MPO (1:2000, ab208670), anti-NF-κB (1:1000, ab32536), anti-NF-κB p65 (1:1000, ab76302), anti-Nrf2 (1:1000, ab92946), anti-NLRP3 (1:1000, ab270449), and anti-iNOS (1:1000, ab178945). After washing three times, the membranes were incubated with a horseradish peroxidase-conjugated secondary antibody (1:10,000, ab6721). The blots were visualized using chemiluminescence detection reagents (Amersham Pharmacia Biotech, Stockholm, Sweden) and Amersham Imager 600 Gel Imaging System (Amersham Pharmacia Biotech). The densitometry analysis of each blot was performed using ImageJ software NIH, USA.

### Statistical analysis

All statistical analyses were performed using GraphPad Prism 8.0.1 software. The results were expressed as mean ± standard error of the mean (SEM). Survival curves of the infected mice were compared using the log-rank (Mantel-Cox) test. Statistical analyses were performed using the repeated measure analysis of variance (ANOVA) (body weight loss) or one-way analysis of variance followed by Tukey’s multiple comparisons test. A value of *p* < 0.05 meant a statistical difference.

## Data Availability

The datasets used and analyzed during the current study are available from the corresponding author on reasonable request.

## Abbreviations

CAT: Catalase
CFU: Colony-forming units
ERK: Extracellular signal-regulated kinases
GI: Gastrointestinal
GSH-Px: Glutathione peroxidase
Hb: Hemoglobin
H&E: Hematoxylin and eosin
HO-1: Heme oxygenase-1
iNOS: Inducible nitric oxide synthase
KC: Keratinocyte-derived chemokine
LPS: Lipopolysaccharide
MCP1: Monocyte chemoattractant protein 1
MDA: Malondialdehyde
MIP2: Macrophage inflammatory protein 2;
MODS: Multiple organ dysfunction syndrome
MPO: Myeloperoxidase
Neu: Neutrophilic granulocyte
Neu%: Percentage of neutrophil
NF-κB: Nuclear factor κB
NLRP3: NOD-like receptor thermal protein domain associated protein 3
Nrf2: Nuclear factor erythroid-derived 2–related factor 2
RBC: Red blood cell
ROS: Reactive oxygen species
SOD: Superoxide dismutase
*S*.Typhimurium: *Salmonella enterica* serovar Typhimurium
TLR: Toll-like receptors
VEGF: Vascular endothelial growth factor
